# Nanocellulose Xerogels With High Porosities and Large Specific Surface Areas

**DOI:** 10.3389/fchem.2019.00316

**Published:** 2019-05-07

**Authors:** Shunsuke Yamasaki, Wataru Sakuma, Hiroaki Yasui, Kazuho Daicho, Tsuguyuki Saito, Shuji Fujisawa, Akira Isogai, Kazuyoshi Kanamori

**Affiliations:** ^1^Department of Biomaterial Sciences, Graduate School of Agricultural and Life Sciences, The University of Tokyo, Tokyo, Japan; ^2^Department of Chemistry, Graduate School of Science, Kyoto University, Kyoto, Japan

**Keywords:** cellulose nanofiber, xerogel, aerogel, porous material, ambient pressure drying

## Abstract

Xerogels are defined as porous structures that are obtained by evaporative drying of wet gels. One challenge is producing xerogels with high porosity and large specific surface areas, which are structurally comparable to supercritical-dried aerogels. Herein, we report on cellulose xerogels with a truly aerogel-like porous structure. These xerogels have a monolithic form with porosities and specific surface areas in the ranges of 71–76% and 340–411 m^2^/g, respectively. Our strategy is based on combining three concepts: (1) the use of a very fine type of cellulose nanofibers (CNFs) with a width of ~3 nm as the skeletal component of the xerogel; (2) increasing the stiffness of wet CNF gels by reinforcing the inter-CNF interactions to sustain their dry shrinkage; and (3) solvent-exchange of wet gels with low-polarity solvents, such as hexane and pentane, to reduce the capillary force on drying. The synergistic effects of combining these approaches lead to improvements in the porous structure in the CNF xerogels.

## Introduction

Aerogels are defined from a processing perspective as porous structures that are obtained by supercritical drying of wet gels (Hüsing and Schubert, 1998). In general, aerogels are mesoporous and combine a high porosity and large specific surface area (SSA). Owing to their specific porous structure, aerogels can excellently function as thermal, acoustic, and electrical insulators as well as catalyst supports, separators, and adsorbents. Some aerogels can be optically transparent yet thermally insulating, and have drawn interest as transparent insulators that might reduce thermal energy loses from windows in offices and automobiles (Hayase et al., 2014; Zu et al., 2018). Aerogels were first reported in 1931 (Kistler, 1931); however, it remains difficult to put aerogels to practical use because of two problems: one is the mechanical brittleness of aerogels; and the other is the process of supercritical drying, which limits productivity and scalability. Therefore, much effort has recently been made to overcome these problems.

In terms of the brittleness, one approach is to use cellulose nanofibers (CNFs) as the skeletal units of aerogels. CNFs are wood-derived emerging materials, which have excellent mechanical strength (~3 GPa) (Saito et al., 2013) and solvent tolerance. CNFs are thus suitable building blocks for aerogels. Pääkkö et al. (2008) demonstrated the flexibility of structurally aerogel-like CNF cryogels, and Kobayashi et al. (2014) have reported on mechanically tough aerogels with good optical transparency and thermal insulating properties, based on a very fine type of CNFs with a width of ~3 nm.

One approach to addressing the processing problem is to switch focus from aerogels to xerogels. Xerogels are defined as porous structures that are obtained by evaporative drying of wet gels. However, producing structurally aerogel-like xerogels under ambient conditions remains particularly challenging. Even thick block-like hydrogels shrink considerably during evaporative drying owing to capillary forces; hence, the resulting xerogels inevitably become thin films with a very low porosity. One approach is to exchange the solvent of the wet gels, which is typically water, to a solvent of lower polarity, such as hexane and pentane, to reduce the capillary force. This concept was reported in a paper by Prakash et al. (1995), in which aerogel-like silica xerogels were formed as a thin film with a high porosity (~99%) through solvent exchange after chemically modifying the silica skeleton to be hydrophobic. Later, Kanamori et al. (2007) further developed the idea and reported that monolithic xerogels can be optically transparent and even exhibit rubbery compression with the use of organosilicons as the skeletal precursor.

In the field of cellulose science, Toivonen et al. (2015) reported a landmark study of CNF xerogels, which were similarly produced through a solvent exchange process. Here CNFs were formed into a wet cake by vacuum filtration of their water dispersion, followed by drying under ambient conditions after solvent exchange with octane. The resulting xerogels were mesoporous and in a film form with a thickness of ~25 μm. Their porosity and SSA were ~60% and 200 m^2^/g, respectively, which are high characteristics among xerogels but still not within the structural range of supercritical-dried CNF aerogels (porosity 60–99%, SSA 300–600 m^2^/g) (Sehaqui et al., 2011; Kobayashi et al., 2014; Sakai et al., 2016). In another route to xerogel formation, freeze-thawing of aqueous CNF dispersions has been used to form block-like hydrogels, followed by solvent exchange. The resulting xerogels were monolithic with a high porosity of over 98% but were macroporous. Their SSA values were ~30 m^2^/g (Li et al., 2017; Erlandsson et al., 2018). Furthermore, a non-CNF skeletal, different type of cellulosic xerogels with a high porosity (~90%) has been produced by a molecular dissolution-regeneration process; however, the SSA values of these materials were ~10–30 m^2^/g (Pour et al., 2016).

Here we report on CNF xerogels with a truly aerogel-like porous structure. The xerogels are in a monolithic form with high porosities and SSA in the ranges of 71–76% and 340–411 m^2^/g, respectively. Our strategy is based on three ideas: (1) the use of a very fine type of CNFs with a width of ~3 nm; (2) increasing the stiffness of precursor hydrogels by reinforcing inter-CNF interactions; and (3) solvent exchange as described above. To our knowledge, these ideas have not yet been combined, and the synergistic effects of these factors lead to the development of porous structure in CNF xerogels.

## Materials and Methods

### Materials

A softwood bleached kraft pulp (Nippon Paper Industries, Japan) was used as the starting sample. The pulp was maintained in an undried state after bleaching. Before use, the pulp was demineralized by stirring in an aqueous HCl solution at pH 2 for 1 h, followed by washing with deionized water. All chemicals and solvents were of laboratory grade (Wako Pure Chemicals, Japan) and used as received.

### Preparation of CNFs

CNF dispersions were prepared according to our previous reports (Saito et al., 2006; Sakai et al., 2016). In brief, the pulp was oxidized by a TEMPO/NaBr/NaClO system with 10 mmol of NaClO added per gram of pulp. The carboxylate content of the oxidized pulp was ~1.6 mmol/g. The oxidized pulp was then suspended in water at a concentration of 0.5% w/w, and disintegrated into CNFs by passing the suspension through a high-pressure water jet system (HJP-25005X, Sugino Machine) five times.

### Xerogel Formation via Acid-Induced Gelation

The 0.5% w/w CNF dispersion (20 g) was poured into a plastic mold with an inner dimension of 6 × 5.5 × 1 cm^3^. A 1 M HCl solution (6 mL) was spread over the dispersion and allowed to stand for 1 h. The resulting hydrogel was removed from the mold and cut into pieces of ~10 × 10 × 5 mm^3^ in size with a sharp blade. Some pieces were immersed into 0.01 M HCl (150 mL) and shaken at ambient conditions for 1 day using an orbital shaker at 50 rpm, followed by rinsing out the HCl with distilled water over 2 days. The hydrogels were then dried at 23°C under ambient pressure. The resulting xerogels are denoted as *w*-xerogels. For reference, supercritical-dried CNF aerogels were also prepared from the same lot of hydrogel according to our previous report (Sakai et al., 2016).

The remaining pieces of the hydrogel were immersed in a mixed solution of 0.01 M HCl (75 mL) and ethanol (75 mL) and shaken under ambient conditions for 1 day using an orbital shaker at 50 rpm. The gel pieces were then shaken in an ethanol bath (280 mL) at 40°C for 3 days, accompanied by replacement of the bath with fresh ethanol twice a day. Some of the alcogel pieces were further shaken in a bath of acetone, hexane or pentane (280 mL) at 40°C for 3 days, accompanied by replacement of the bath with each fresh solvent twice a day. Each of the ethanol-, acetone-, hexane-, and pentane-exchanged gels was dried at 23°C under ambient pressure in an airtight desiccator containing silica gel at the bottom; the resulting xerogels are denoted as *e*-, *a*-, *h*- and *p*-xerogels, respectively.

### Xerogel Formation via Metal Ion-Induced Gelation

The 0.5% w/w CNF dispersion (20 g) was poured into the same plastic mold as for that used for the acid-induced gelation (see section Xerogel Formation via Acid-Induced Gelation). A 0.1 M solution of MgCl_2_, CaCl_2_, or AlCl_3_ (6 mL) was spread over the dispersions, which were then allowed to stand for 24 h. Similar to the case of the acid-induced gelation, the resulting hydrogel was cut into pieces with dimensions of ~10 × 10 × 6 mm^3^. The gel pieces were shaken in a 0.01 M solution of CaCl_2_, MgCl_2_, or AlCl_3_ (150 mL) for 1 day and then successively in a mixed solution of water (75 mL) and ethanol (75 mL) for 1 day each. The subsequent processing after solvent exchange with the mixed solutions involved the same steps as for the acid-induced gelation with ethanol and hexane. The xerogels resulting from evaporative drying of hexane are denoted as *h*-*Mg*-, *h*-*Ca*-, and *h*-*Al*-xerogels.

### Membrane Xerogel Formation

An 0.5% w/w CNF dispersion (27 g) was poured into a glass dish with an inner diameter of 7 cm. A 0.1 M AlCl_3_ solution (8 mL) was spread over the dispersion, which was then allowed to stand for 24 h. The originally air-exposed, top surface of the resulting hydrogel was cut off using a sharp blade to obtain a columnar gel with a thickness of ~6 mm. The shaped gel was shaken in a 0.01 M AlCl_3_ solution (150 mL) for 1 day and successively in a mixed solution of water (75 mL) and ethanol (75 mL) for 1 day each. The subsequent process involved the same solvent-exchange steps with the use of ethanol and hexane for the acid-induced gelation. The hexane-exchanged gel was then sandwiched with flat PTFE plates and allowed to dry at 23°C under ambient pressure in an airtight desiccator containing silica gel at its bottom. The top PTFE plate was 34 g in weight.

### Analyses

Scanning electron microscope (SEM) images were acquired with a Hitachi S-4800 field-emission microscope operated at 1 kV. Prior to SEM observations, the xerogels were split using tweezers, and the exposed cross-sections were thinly coated with osmium with the use of a Meiwafosis Neo osmium coater at 5 mA for 10 s. Nitrogen adsorption–desorption isotherms were measured with a Quantachrome NOVA 4200e at −196°C after degassing of the samples in the system at 105°C for 3 h. The SSA values and pore size distributions of the xerogels were estimated from the isotherms following Brunauer–Emmett–Teller (BET) theory and the Barrett–Joyner–Halenda (BJH) model, respectively. The porosity values (%) of the xerogels were calculated using the following equation,

$$\begin{array}{l} Porosity=\frac{V_{p}}{V_{p}+1/\rho_{t}}\times \;100, \end{array}$$

where *V*_*p*_ is the pore volume (cm^3^/g) of the corresponding xerogel, and ρ_*t*_ is the true density of the CNF (1.64 g/cm^3^). The pore volumes *V*_*p*_ of the xerogels were estimated from the maximum amounts of N_2_ adsorbed into the respective xerogels by converting the adsorbed amount expressed as a gas volume at 273 K and 1 atm into pore-filling volumes as liquid nitrogen at −196°C. The porosity of the reference aerogel was calculated in the usual manner from its apparent volume and weight. Fourier transform infrared (FTIR) spectra of the xerogels were obtained with a JASCO FT/IR-6100. Tensile testing of the membranous xerogel was performed at 23°C and 50% relative humidity with the use of a Shimadzu EZ-TEST equipped with a 500 N load cell. The specimens were ~2 mm wide and 20 mm long. Ten specimens were measured with a span length of 10 mm at a rate of 1.0 mm/min. The thermal conductivity *k* of the membranous xerogel was estimated based on the following equation,

$$\begin{array}{l} k=\;\rho \alpha c, \end{array}$$

where ρ, α and *c* are the bulk density, thermal diffusivity, and specific heat capacity of the xerogel, respectively. The thermal diffusivity α (9.02 × 10^−8^ m^2^/s) was measured at 23°C and 50% relative humidity using an ai-Phase Mobile 1U device. The specific heat capacity *c* at 23°C (1.23 J/g·K) was measured using a Perkin-Elmer DSC 8500 instrument.

## Results and Discussion

The CNF in the present study was chemically modified by a TEMPO-oxidation process (see section Materials and Methods). The TEMPO-oxidized CNFs are characterized by a uniform width of ~3 nm (Daicho et al., 2018), and by a high density of surface carboxyl groups (1–1.7 groups/nm^2^). The carboxyl groups are in the form of sodium salts and are well-dissociated in water. Accordingly, electric double layer repulsion is induced between the CNFs in water, and these CNFs are stably dispersed. Their dispersibility strongly depends on the pH of the system and the presence of co-existing salts (Tanaka et al., 2014, 2016). The CNFs start to attractively interact by lowering of the pH below the p*K*_a_ value 3.6 for the carboxyl group and form a stiff hydrogel (Saito et al., 2011). Here, the sodium carboxylates (-COO^−^Na^+^) turn into carboxylic acids (-COOH). The inter-CNF repulsion is lost, and the CNFs are partially assembled or networked through hydrogen bonding and other van der Waals interactions. For this sol-gel transition, the storage modulus G′ of the system increases by several orders of magnitude. Similar behavior is observed when multivalent metal ions, such as Ca^2+^ and Al^3+^, are added into the system (Goi et al., 2018). The inter-CNF interactions are dominated by ionic bonds in this case, such that the resulting hydrogels are thereby stiffer than the acid-type gels.

### Xerogels via Acid-Induced Gelation

**Figure 1:**
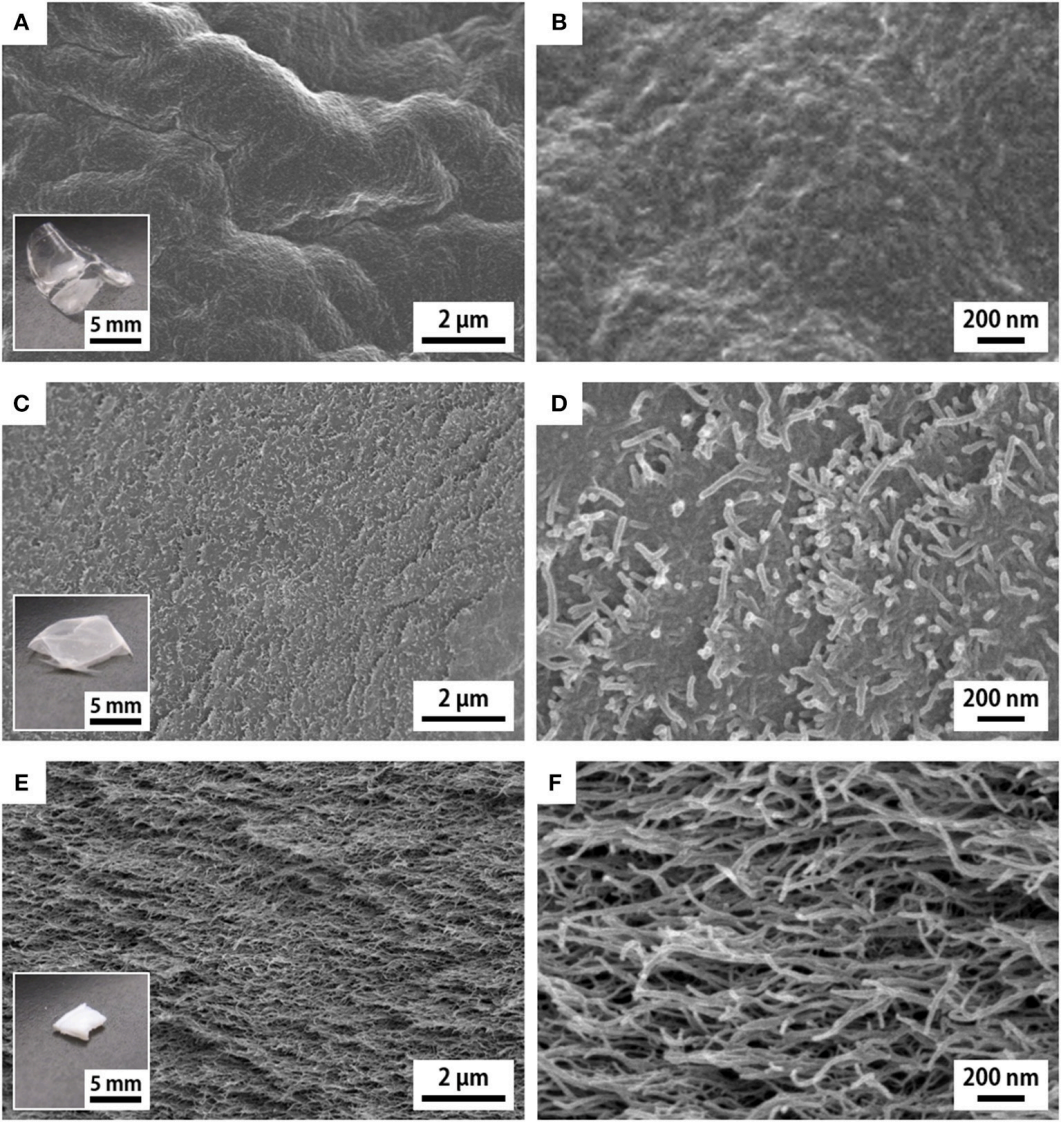
SEM images of the cross sections of the **(A,B)** *w*-xerogel, **(C,D)** *a*-xerogel, and **(E,F)** *h*-xerogel. Insets show the appearances of the respective xerogels.

Initially, the acid type hydrogels were solvent-exchanged with ethanol, acetone, hexane, and pentane, followed by evaporative drying at 23°C under ambient pressure. For reference, some of the hydrogel samples were directly subjected to evaporative drying. Although all the hydrogels were shaped in block-like pieces with a size of ~10 × 10 × 6 mm^3^ (Supplementary Figure S1), the resulting xerogels through solvent exchange differed from each other in both appearance and skeletal structure (Figure 1).

The reference *w*-xerogels, obtained by evaporation of water, formed thin and optically transparent films (the inset of Figure 1A). The CNF morphology was invisible at the cross sections of the *w*-xerogel films by SEM (Figures 1A,B). We explain this result by the close assembly of CNFs, which was induced by the strong capillary force of water (surface tension γ ≈ 72 mN/m). The *e*- and *a*-xerogels obtained by evaporation of ethanol (γ ≈ 22 mN/m) and acetone (γ ≈ 24 mN/m) also formed thin films but had a hazy appearance (the inset of Figure 1C). The CNF morphology at their cross sections became visible, although the CNFs remained closely assembled (Figures 1C,D). Ethanol and acetone are also likely to interact strongly with the polar groups on the CNF surfaces, such as hydroxyl and carboxyl groups, resulting in the close assembly of CNFs at a meniscus on the solvent evaporation. Notable cases were found for the *h*- and *p*-xerogels, which were obtained by evaporation of hexane (γ ≈ 18 mN/m) and pentane (γ ≈ 16 mN/m), respectively. These xerogels had a block-like, monolithic shape (~1 mm thick) and were optically opaque (the inset of Figure 1E). The CNF assembly on drying was sufficiently suppressed, such that a network-like CNF skeleton was formed within the xerogels (Figures 1E,F). There might exist a threshold at ~γ ≈ 20 mN/m for the solvent developing a porous structure within the xerogels, in the case when no further hydrophobization or stronger cross-linking was applied to the TEMPO-oxidized CNFs.

**Figure 2:**
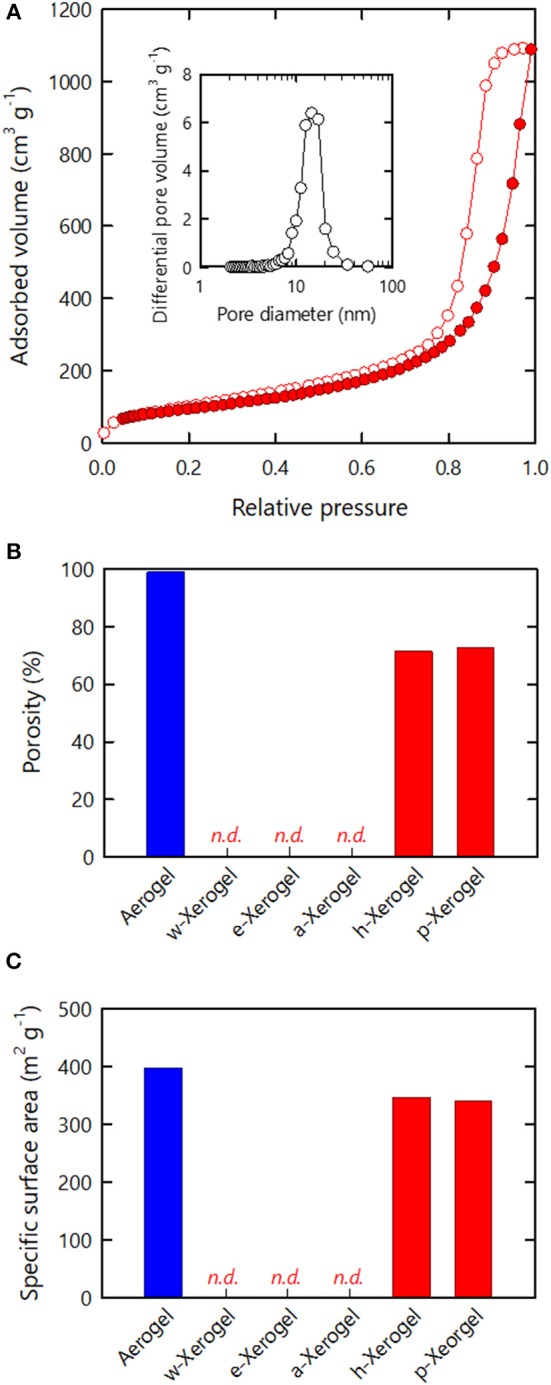
Nitrogen adsorption–desorption analyses of the xerogels. **(A)** Isotherm of the *h*-xerogel. Inset shows the pore size distribution estimated from the isotherm. **(B)** Porosity and **(C)** SSA values estimated from the isotherms of the respective xerogels.

Nitrogen adsorption–desorption analyses were performed on the series of xerogels (Figure 2). For reference, we also analyzed aerogels obtained from the acid type CNF hydrogels via supercritical drying (Supplementary Figure S2). Although the *w*-, *e*- and *a*-xerogels were non-porous, as shown in Figure 1, and could not be analyzed, both the *h*- and *p*-xerogels exhibited typical isotherms of mesoporous structures (Figure 2A). The isotherms were characterized by hysteresis at a high relative pressure, caused by capillary condensation of nitrogen gas in pores. The pore sizes estimated from the isotherms of the *h*- and *p*-xerogels ranged from a few nm to ~30 nm (the inset of Figure 2A). Note that the capillary pressures on nitrogen sorption can be of order 1 MPa (Reichenauer and Scherer, 2001) and thus shrinkage of the xerogels should be inevitable at a high relative pressure; the pore sizes in the present study are lower estimates.

The porosity values of the xerogels were also estimated from the isotherms (Figure 2B), where the maximum amounts of nitrogen adsorbed were regarded as the pore filling volumes of liquefied nitrogen through capillary condensation (see section Materials and Methods). The resulting values thus reflect the lower limit of the porosity. Although the porosity of materials is commonly evaluated from their dimensions, mass, and true density, in the present study it was difficult to reliably measure the dimensions of the series of xerogels, which had distorted shapes and rough surfaces. The porosities of the *w*-, *e*-, and *a*-xerogels, evaluated on this basis, were below the limit of detection. The porosity values of the *h*- and *p*-xerogels were found to be as high as 70%. A value of 70% is within the porosity range of CNF aerogels reported to date (>60%) (Sehaqui et al., 2011; Sakai et al., 2016), but was lower than the porosity of the reference aerogel, as shown in Figure 2B (~99%). Note that the reference aerogels in the present study were prepared by acid-induced gelation of TEMPO-oxidized CNFs; their structural profiles as porous materials, including porosity and SSA, are among the highest reported for cellulosic porous structures (Kobayashi et al., 2014).

The SSA results of the xerogels are shown in Figure 2C. Similar to the porosity, the SSA values of the *w*-, *e*- and *a*-xerogels were below the limit of detection (~2 m^2^/g). The SSA values of both the *h*- and *p*-xerogels were as high as 340 m^2^/g. Although the value of 340 m^2^/g was lower than the SSA of the reference aerogels (~400 m^2^/g), it is the highest SSA value ever reported for an ambient pressure-dried xerogel of cellulose or its derivatives. These results justify our combined strategy of: 1) the use of 3 nm-wide TEMPO-oxidized CNFs; 2) stiffening the inter-CNF interaction; and 3) solvent exchange.

### Xerogels via Metal Ion-Induced Gelation

We examined further reinforcing the inter-CNF interactions by ionically cross-linking carboxylate groups via multivalent metal ions, namely Mg^2+^, Ca^2+^, and Al^3+^ (Goi et al., 2018). The resulting hydrogels were solvent-exchanged with hexane based on the results in the previous section, followed by evaporative drying at 23°C under ambient pressure.

**Figure 3:**
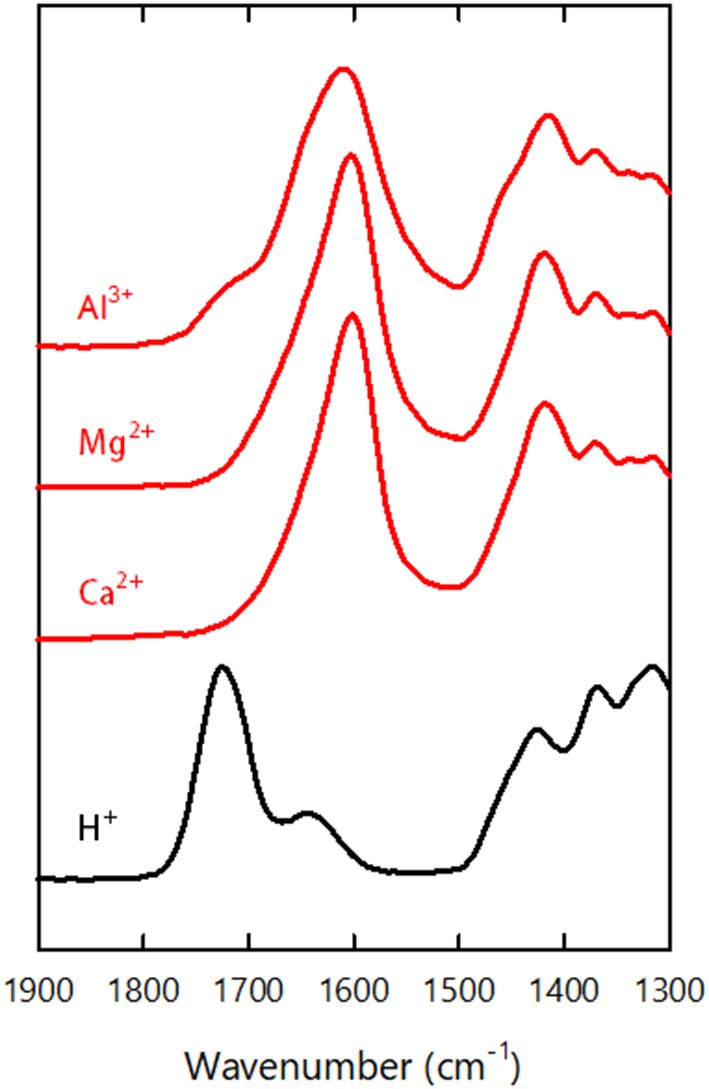
FTIR spectra of the *h*-xerogel, *h*-*Ca*-xerogel, *h*-*Mg*-xerogel, and *h*-*Al*-xerogel.

Figure 3 shows FTIR spectra of the xerogels formed through metal ion-induced gelation (*h*-*Mg*-, *h*-*Ca*-, and *h*-*Al*-xerogels). For reference, the spectrum of the acid-type one (*h*-xerogel) is also shown. In the spectra, the carboxyl-related IR adsorptions were as follows (Fujisawa et al., 2011): the C = O stretch of the carboxylic acid groups was at 1,720 cm^−1^, the C = O stretch of carboxylate groups was at 1,600 cm^−1^, and the C–O symmetric stretching of carboxylate groups was at 1,410 cm^−1^. An adsorption peak was also present from bending of adsorbed water molecules at 1,630 cm^−1^. The reference *h*-xerogel showed no peaks at 1,600 and 1,410 cm^−1^ but had a clear adsorption at 1,720 cm^−1^. Meanwhile, the *h*-*Mg*-, *h*-*Ca*-, and *h*-*Al*-xerogels showed little or no adsorption at 1,720 cm^−1^ but had distinct peaks at 1,600 and 1,410 cm^−1^, regardless of the ionic species present. The small adsorption at 1,720 cm^−1^ for the *h*-*Al*-xerogel indicated the presence of carboxylic acid groups. This result is explained by the pH of the gelling agent; a 0.01 M AlCl_3_ solution that has a pH value of 3.8 was used in the gelation process for the *h*-*Al*-xerogel, and some of the carboxyl groups will be protonated because their p*K*_a_ value is 3.6. We estimated the ratio of the carboxylic acid groups to the total carboxylate content to be ~15% based on the peak area. For *h*-*Mg*- and *h*-*Ca*-xerogels, MgCl_2_ and CaCl_2_ solutions with pH values of ~6 were used, such that no protonation occurred in the gelation process. Furthermore, neither Na^+^ ions in the starting CNF dispersion nor Cl^−^ ions in the gelling agents were detected by energy-dispersive X-ray (EDX) spectroscopy (data not shown). In fact, each multivalent metal ion in the xerogel electrostatically interacted with two or three carboxylate anions on the same or two different CNF surfaces. Otherwise, hydroxide ions would be coupled to electrically neutralize the remaining charges of the metal salts of carboxyl groups, for example, -COO^−^ Al(OH)${}_{2}^{+}$.

**Figure 4:**
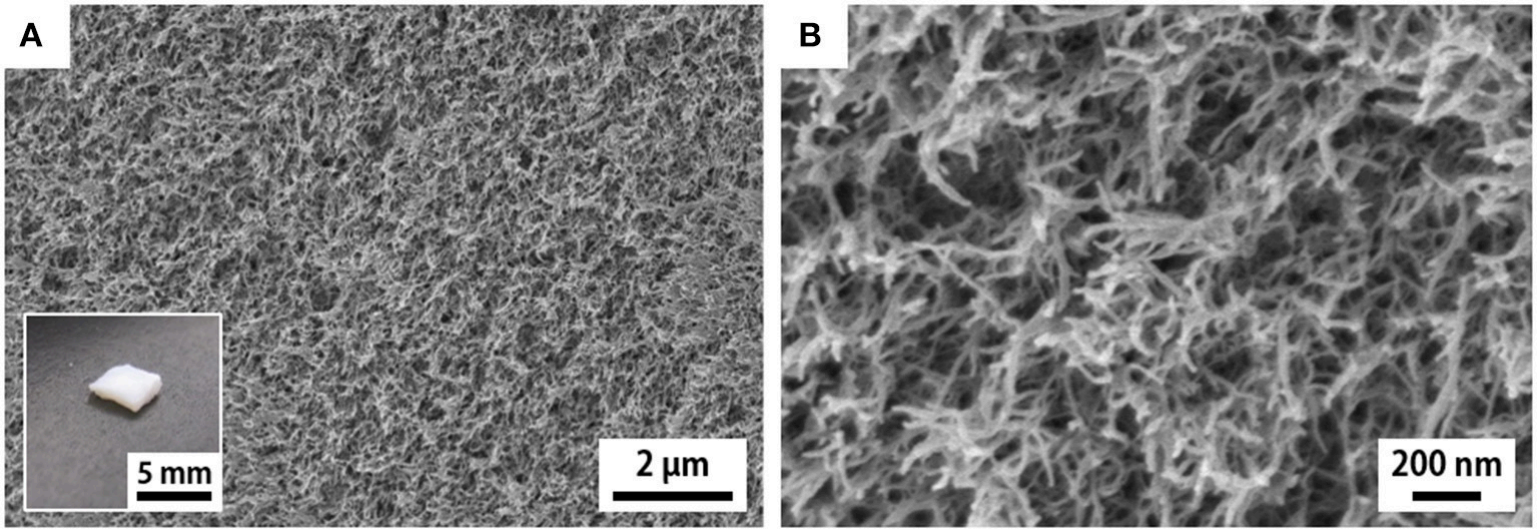
SEM images of the cross section of the *h*-*Al*-xerogel. **(A,B)** show the images taken at low and high magnifications, respectively. Inset shows appearance of the *h*-*Al*-xerogel.

**Figure 5:**
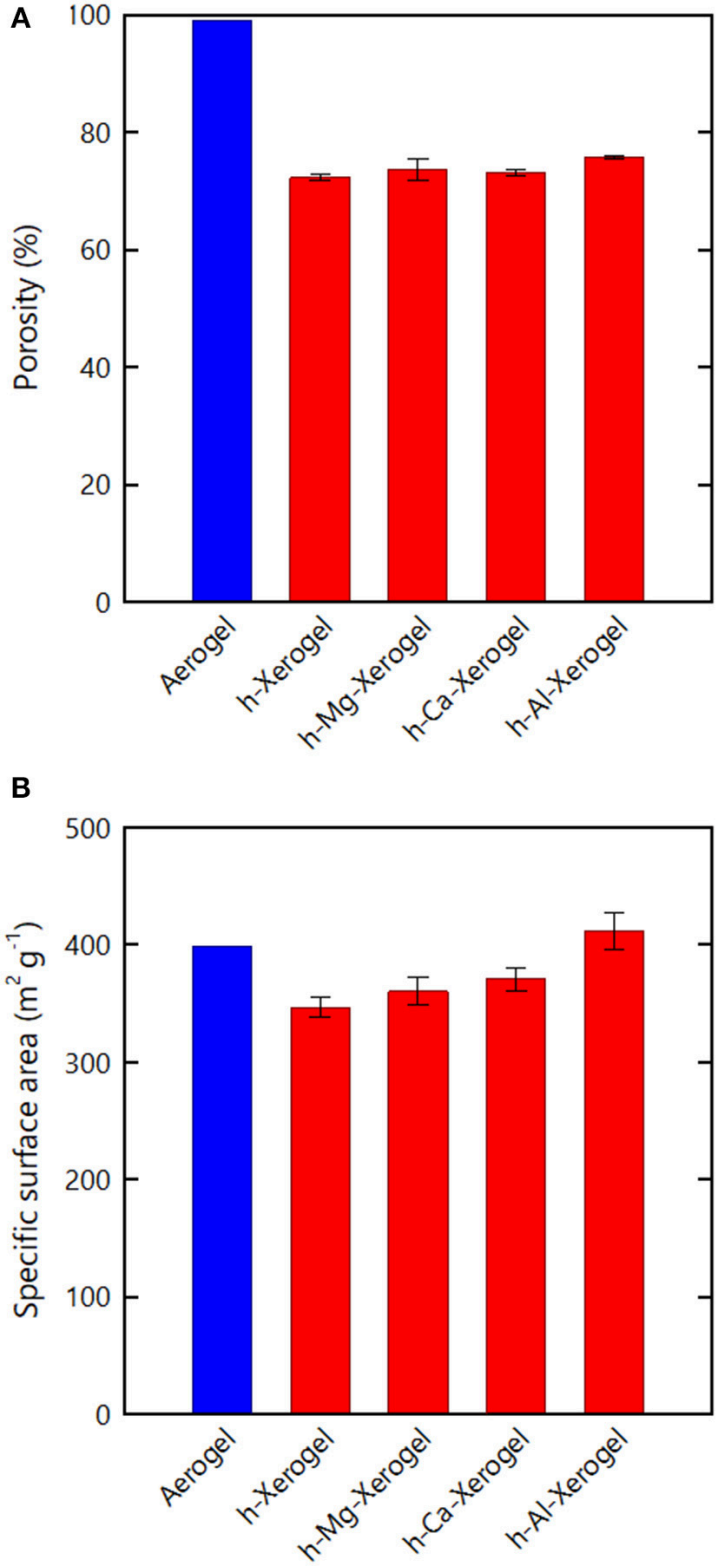
**(A)** Porosity and **(B)** SSA values estimated from the nitrogen adsorption–desorption isotherms of the respective xerogels.

As for the case of the *h*-xerogels, all the *h*-*Mg*-, *h*-*Ca*-, and *h*-*Al*-xerogels possessed a monolithic shape (roughly 1 mm in thickness), and a network-like CNF skeleton formed within the xerogels (Figure 4, Supplementary Figure S3). These xerogels showed similar nitrogen adsorption–desorption isotherms to those for the *h*-xerogels (see Figure 2A). The porosity values, which were estimated from the isotherms, increased slightly from 71% for the *h*-xerogel to 76% for the *h*-*Al*-xerogel (Figure 5A). The SSA significantly increased from a high value of ~340 m^2^/g for the *h*-xerogel to 410 m^2^/g for the *h*-*Al*-xerogel (Figure 5B). This value of 410 m^2^/g is truly comparable to the SSA of the reference aerogels (~400 m^2^/g). Considering the electrostatic interactions with the carboxylate anions, the trivalent ions of Al^3+^ were likely more effective than the divalent ions of Mg^2+^ and Ca^2+^, in improving the porosity and SSA.

### Membrane Xerogels

**Figure 6:**
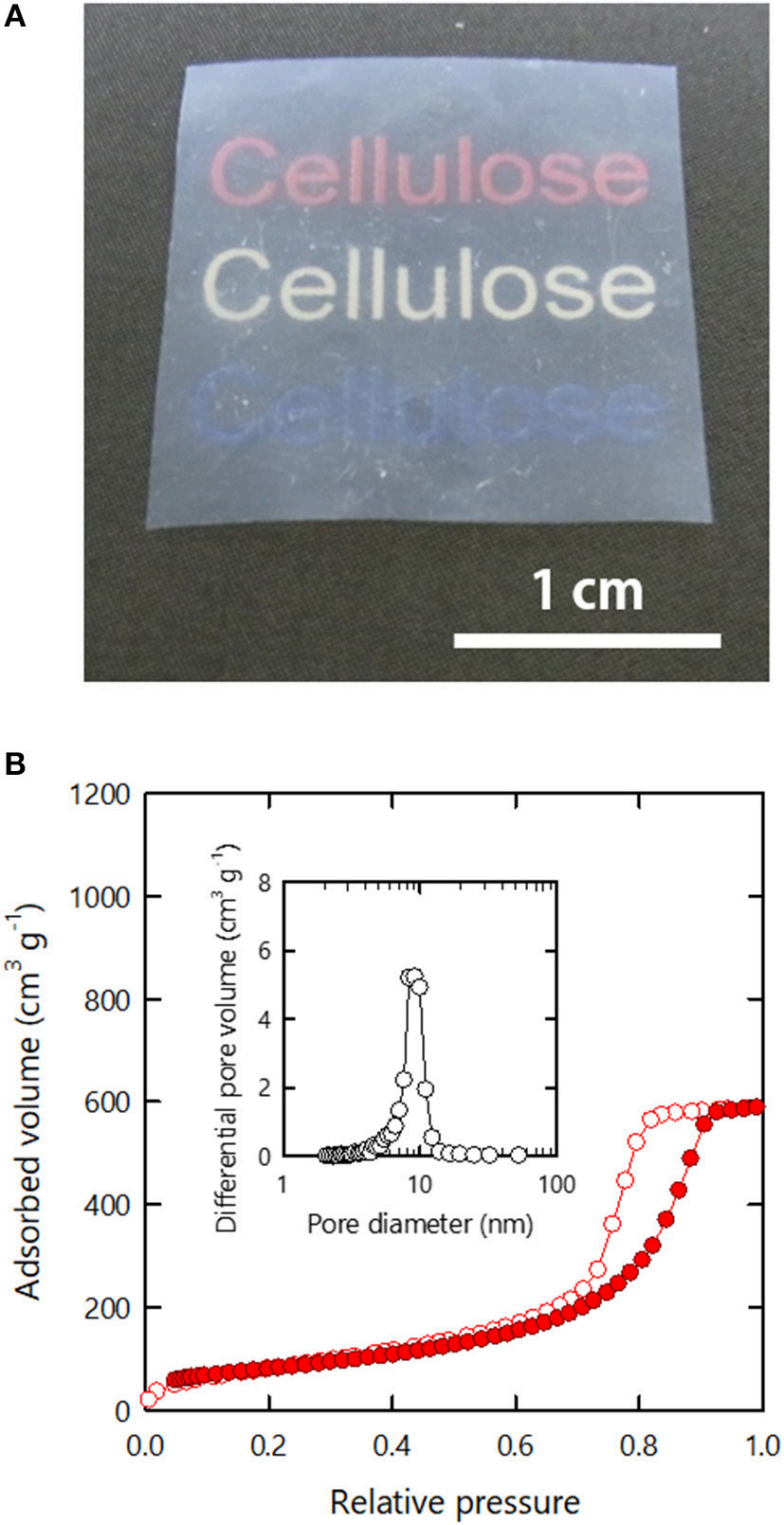
**(A)** Appearance and **(B)** nitrogen adsorption–desorption isotherm of the membranous *h*-*Al*-xerogel. Inset in **(B)** shows the pore size distribution estimated from the isotherm.

The *h*-*Al*-xerogels were optically opaque as shown in Figure 4, despite their high porosity and SSA. We attribute this result to their surface roughness. Thus, we tried to reduce the surface roughness of the *h*-*Al*-xerogels by sandwiching the precursor organogels with flat PTFE plates during drying (see section Materials and Methods). The compression force imposed on the organogels by sandwiching was estimated to be ~87 Pa. Although the resulting *h*-*Al*-xerogels formed films (~74 μm), their optical transmittance improved greatly (Figure 6A) (see Supplementary Figure S4 for a light transmittance spectrum). In addition, the nitrogen adsorption–desorption isotherms of the film-like *h*-*Al*-xerogels were as categorized as type IV IUPAC physisorption, similar to the monolithic *h*- and *h*-*Al*-xerogels (Figure 6B). The porosity and SSA values were then estimated from the isotherm to be ~60% and 270 m^2^/g, respectively. These values are lower than those of the *h*-*Al*-xerogels but still high as those of cellulosic xerogels.

Unlike the other xerogels in the present study, the film-like *h*-*Al*-xerogels had sufficiently flat surfaces that their specimen sizes could be reliably measured. Next, we analyzed the tensile properties and thermal conductivity of the film-like *h*-*Al*-xerogels. In the tensile tests, the film-like xerogels exhibited a high Young's modulus *E* and tensile strength σ of ~3 and 49 MPa, respectively (see Supplementary Figure S5 for a typical stress–strain curve). These values are within the range of the *E* and σ values of practically applicable plastic films (Ashby et al., 2009), which is surprising because of the high porosity and a large SSA of the xerogels. We explain this result by the high strength of the CNFs and their strong interactions at the contacting surfaces. The thermal conductivity *k* of the film-like xerogels was measured to be 0.095 W/m·K in the out-of-plain direction. This value is intermediate between those of highly-compacted CNF films (0.11–0.14 W/m·K) (Zhao et al., 2018) and insulating CNF aerogels (0.02–0.04 W/m·K) (Sakai et al., 2016). The porosity and SSA of the xerogels (~60% and 270 m^2^/g) were both higher than those of the compacted films (10–18% and < 2 m^2^/g) but lower than those of the insulating aerogels (>97% and > 300 m^2^/g). Therefore, the drying conditions should be further explored to obtain heat-insulating yet optically-transparent CNF xerogels with a truly aerogel-like porous structure.

## Conclusions

Monolithic CNF xerogels with high porosities and large SSAs in the ranges of 71–76% and 340–411 m^2^/g, respectively, were prepared via evaporative drying under ambient conditions. These characteristics fall within the structural range of supercritical-dried CNF aerogels reported to date. These materials were achieved by a combination of three concepts: (1) the use of TEMPO-oxidized CNFs with a uniform width of ~3 nm to exploit the potential SSA of CNFs; (2) stiffening the inter-CNF interactions in the hydrogels by, for example, ionic cross-linking to withstand their dry shrinkage; and (3) solvent exchange with low-polarity solvents such as hexane and pentane to reduce the capillary forces during drying. All the monolithic xerogels had distorted shapes and rough surfaces. Accordingly, these materials were opaque despite having high porosity and SSA. Therefore, further exploration of the drying conditions might enable monolithic yet optically-transparent CNF xerogels. Such trials should also look to optimize the inter-CNF interactions and make the CNF surfaces more hydrophobic.

## Data Availability

The datasets for this manuscript are not publicly available because the raw data supporting the conclusions of this manuscript will be made available by the authors, without undue reservation, to any qualified researcher. Requests to access the datasets should be directed to TS, asaitot@mail.ecc.u-tokyo.ac.jp.

## Author Contributions

TS conceived the concept of the study. SY and TS designed the experiments with the help of KK. SY performed most of the experiments with the help of WS, HY, and KD. All the authors analyzed the data. SY and TS mainly wrote the manuscript with the contributions of all the authors.

### Conflict of Interest Statement

The authors declare that the research was conducted in the absence of any commercial or financial relationships that could be construed as a potential conflict of interest.
