# Supplementary material for: Nanocellulose Xerogels With High Porosities and Large Specific Surface Areas
Source: Front Chem. 2019 May 7;7:316. doi: 10.3389/fchem.2019.00316 (PMC6514048; doi:10.3389/fchem.2019.00316)
Supplement: Supplementary file 1 [file Data_Sheet_1.PDF]

Supplementary Material

Nanocellulose Xerogels with High Porosities and Large Specific Surface Areas

Shunsuke Yamasaki, Wataru Sakuma, Hiroaki Yasui, Kazuho Daicho, Tsuguyuki Saito*, Shuji Fujisawa, Akira Isogai, and Kazuyoshi Kanamori

*** Correspondence:** Tsuguyuki Saito: asaitot@mail.ecc.u-tokyo.ac.jp

This file includes **Supplementary Figures S1–S5**.

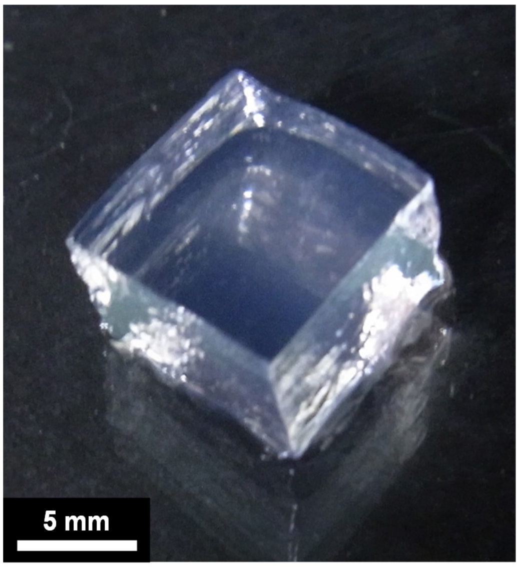

Supplementary Figure S1. Appearance of a hydrogel piece.

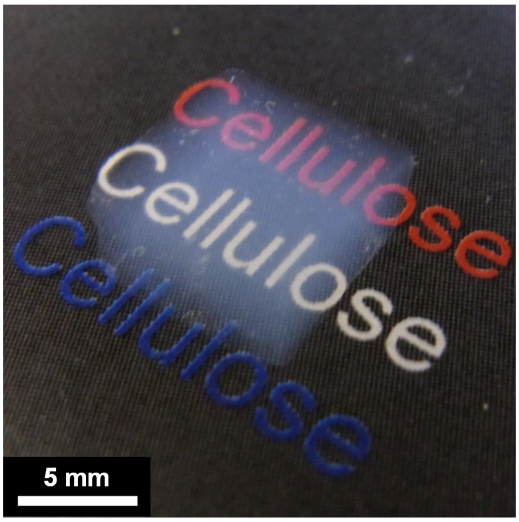

Supplementary Figure S2. Appearance of the reference CNF aerogel.

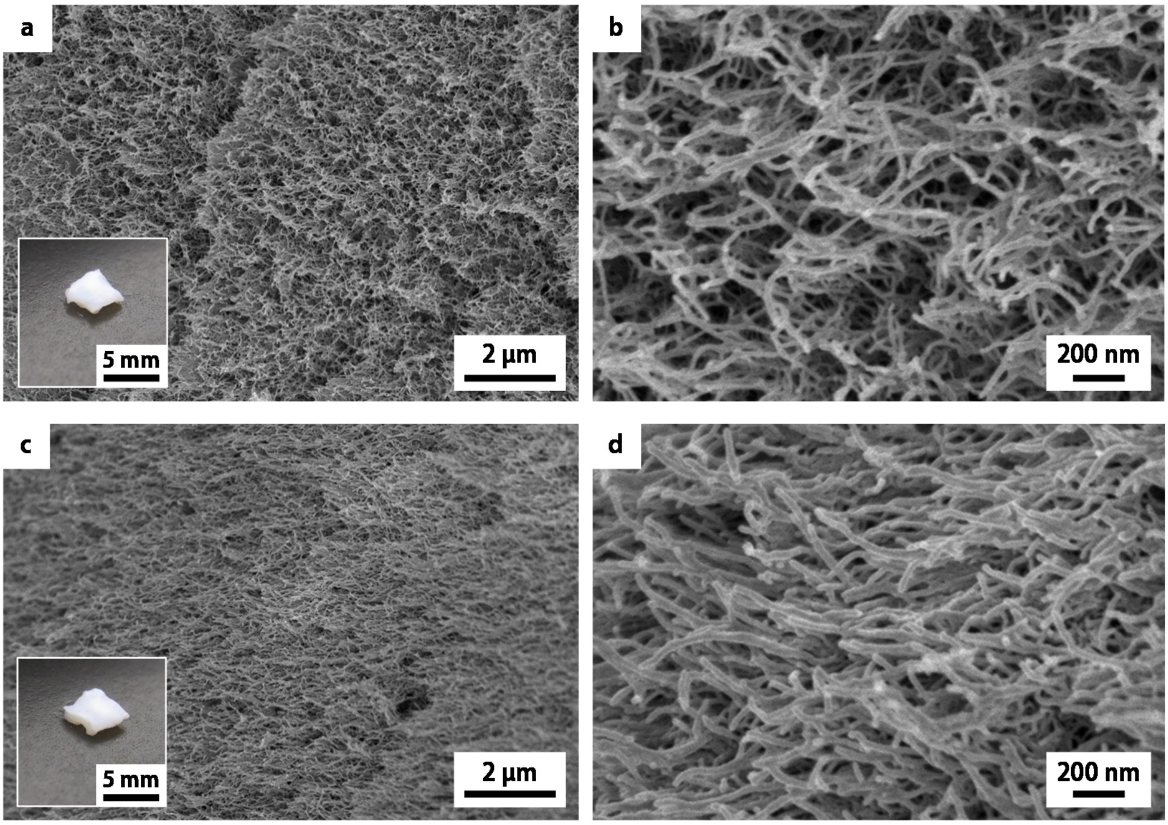

Supplementary Figure S3. SEM images of the cross sections of the (a,b) h-Ca-xerogel and (c,d) h-Mg-xerogel. Insets show the appearances of the respective xerogels.

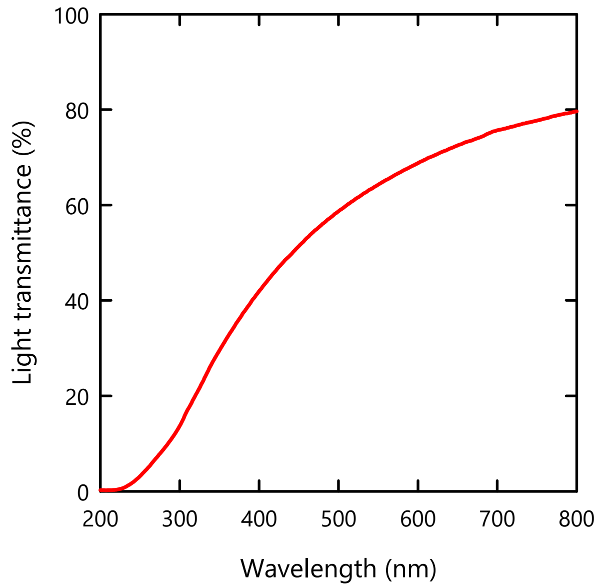

Supplementary Figure S4. Light transmittance spectrum of the membrane h-Al-xerogel.

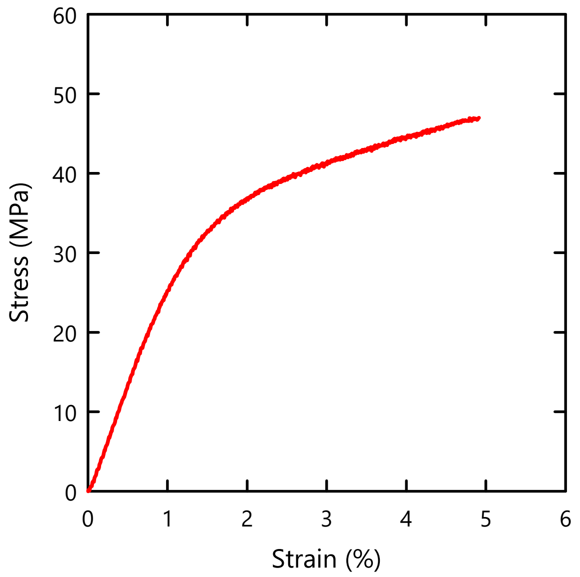

Supplementary Figure S5. Tensile stress–strain curve of the membrane h-Al-xerogel.
